# Exploration of the Antioxidant and Anti-inflammatory Potential of *Cassia sieberiana* DC and *Piliostigma thonningii* (Schumach.) Milne-Redh, Traditionally Used in the Treatment of Hepatitis in the Hauts-Bassins Region of Burkina Faso

**DOI:** 10.3390/ph16010133

**Published:** 2023-01-16

**Authors:** Eliasse Zongo, Anna Busuioc, Roland Nâg-Tiero Meda, Andreea Veronica Botezatu, Maria Daniela Mihaila, Ana-Maria Mocanu, Sorin Marius Avramescu, Benjamin Kouliga Koama, Sami Eric Kam, Hadidiatou Belem, Franck Le Sage Somda, Clarisse Ouedraogo, Georges Anicet Ouedraogo, Rodica Mihaela Dinica

**Affiliations:** 1 Laboratoire de Recherche et d’Enseignement en Santé et Biotechnologies Animales, Université Nazi BONI, Bobo Dioulasso 01 BP 1091, Burkina Faso; 2 Department of Chemistry, Physics and Environment, Faculty of Sciences and Environment, “Dunărea de Jos” University of Galati, 111 Domnească Street, 800201 Galati, Romania; 3 Department of Organic Chemistry, Biochemistry and Catalysis, Faculty of Chemistry, University of Bucharest, 90-92 Soseaua Panduri, 050663 Bucharest, Romania

**Keywords:** anti-inflammatory, antioxidants, *Cassia sieberiana*, *Piliostigma thonningii*

## Abstract

Inflammation is the supreme biological response to illness. In the Hauts-Bassins region, in traditional medicine, all parts of *Cassia sieberiana* and *Piliostigma thonningii* are used to treat hepatitis and inflammation. The aim of this study was to evaluate the in vitro antioxidant and anti-inflammatory activities of their aqueous extracts. High performance liquid chromatography with photodiode array (HPLC-DAD) and ultra-high-performance liquid chromatography coupled with high-resolution mass spectrometry (UHPLC-MS/MS) analyses highlighted the presence of polyphenols and flavonoids. Antioxidant and anti-inflammatory activities were measured by various methods such as DPPH (2,2-diphenyl-1-picrylhydrazyl), ABTS 2,2’-azino-bis(3-ethylbenzothiazoline-6-sulfonic acid), TAC (total antioxidant capacity), anti-protease, anti-lipoxygenase, and membrane stabilization. The best antioxidant activity was observed in the bark (DPPH: IC50 = 13.45 ± 0.10 µg/mL) and roots (TAC = 29.68 ± 1.48 mg AAE/g DW) of *Piliostigma thonningii* and in the roots (ABTS: IC50 = 1.83 ± 0.34 µg/mL) of *Cassia sieberiana*. The best anti-inflammatory activity was observed in the bark (anti-lipoxygenase: IC50 = 13.04 ± 1.99 µg/mL) and leaves (anti-proteases: IC50 = 75.74 ± 1.07 µg/mL, membrane stabilization: IC50 = 48.32 ± 6.39 µg/mL) of *Cassia sieberiana*. Total polyphenols (ABTS: r = −0.679, TAC: r = 0.960) and condensed tannins (ABTS: r = −0.702, TAC: r = 0.701) were strongly correlated with antioxidant activity. Total flavonoids (anti-proteases: r = −0.729), condensed tannins (anti-proteases: r = 0.698), and vitamin C (anti-proteases: r = −0.953) were strongly correlated with anti-inflammatory activity. Total polyphenols, flavonoids, condensed tannins, and vitamin C could contribute to the antioxidant and anti-inflammatory activities of the two studied plants. These results could validate the traditional use of these plants to treat various inflammatory diseases.

## 1. Introduction

Inflammation refers to a series of biological reactions of vascular tissues subjected to stimuli with harmful effects, and is a complex process associated with pain, increased vascular permeability, changes in the membrane structure, and protein denaturation [1]. The causes of the inflammation can be multiple in response to damaged cells of the body, caused by either microbes or by physical or chemical agents [1,2]. Mediators of inflammation include oxygenated free radicals, which maintain an inflammatory state [3,4]. The close relationship between oxidative stress and inflammation may justify the use of molecules with antioxidant effects for anti-inflammatory treatments [5].

However, nonsteroidal anti-inflammatory drugs (NSAIDs) are among the most used drugs worldwide [6]. The association of NSAID use with many side effects on the gastrointestinal tract, kidneys, and liver is considered a major problem in the administration of these drugs in various therapies [7,8,9]. Due to the major side effects related to treatment with NSAIDs, there is a growing interest in natural herbal remedies. In this context, several researchers have shown a growing interest in research and development on the evaluation of herbal medicines as a better treatment of anti-inflammatory disorders [10,11]. 

The chemical composition of plants is varied, some of the most common components being polyphenols, tannins, and terpenes [10,11]. Many recent studies have shown that polyphenols and flavonoids play an important role in preventing chronic diseases such as hepatitis, cardiovascular disease, diabetes, various inflammations, and even cancer [12,13,14]. These classes of compounds exhibit several biological activities [15], and are in particular renowned for their antioxidant properties [16,17]. However, the chemical structure of each phytoconstituent can make a difference in the mechanism of antioxidant and anti-inflammatory activities [18].

Ethnobotanical studies carried out in the Hauts-Bassins region of Burkina Faso have reported that the decoction of all parts of *Cassia sieberiana* and *Piliostigma thonningii* species, reputed in African traditional medicine, are used by herbalists to treat hepatitis, malaria, ulcers, and other inflammatory pathologies [19,20,21]. 

*Cassia sieberiana* DC (*C. sieberiana*), from the Fabaceae family, is widespread in the southern Sahel and Sudan savannah in West and Central Africa [22]. *Piliostigma thonningii* (Schumach.) Milne-Redh (*P. thonningii*) (homotypic synonym: *Bauhinia thonningii* Schumach.) is a legume belonging to the same plant family and is widespread in Africa and Asia [23,24]. These species are widely used in traditional medicine in their local area [10].

The literature data have shown rich and varied compositions in fruits (pulp) of *C. sieberiana* and leaves of *P. thonningii,* with compounds that belong to the classes of tannins, alkaloids, saponins, steroids, flavonoids, cardiac glycosides, cyanogenic glycosides, and sugars [25,26], imparting important therapeutic properties to these compounds. Hydroethanolic extracts of *C. sieberiana* contain rhein derivatives, anthraquinone compounds especially employed for the treatment of incurable diseases such as arthritis, diabetic nephropathy, and cancer due to their unique mechanism of action [19]. The anthelmintic properties of the root bark of *C. sieberiana* and *Piliostigma thonningii* are attributed to their extracts rich in condensed tannins, flavonoids, and polyols such as D-pinitol, a natural polyol, which also support their ethnopharmacological use [27,28]. The presence of various metabolites in different extracts of *P. thonningii* root bark, such as hydroxybenzoic acid derivatives, lignans (α-conidendrin), or dipeptides (anabellamide), prove its pharmacological potential through antioxidant and anti-inflammatory activities by activation of NRF2 [29].

The specific pharmacological properties of *C. sieberiana* species are anti-inflammatory, antinociceptive, muscle relaxant, antispasmodic, antioxidant, gastric cytoprotective, and laxative [30]. The leaves and fruits of *P. thonningii* can protect against the accumulation of cholesterol and triglycerides in the blood [31] but can also have antioxidant and anti-malarial activities [32]. In addition to antioxidant and anti-inflammatory activities, these two species have common antiviral and antibacterial properties [30,33]. 

Most of the plant studies mentioned above were conducted on native species in Nigeria. To date, to the best of our knowledge, no studies have been reported on *C. sieberiana* and *P. thonningii* species in the Hauts-Bassins region of Burkina Faso, where the decoctions of these plants have been used for a long time due to their different therapeutic properties. Therefore, the aim of this research was to analyze the chemical composition of aqueous extracts from different parts of *C. sieberiana* and *P. thonningii* to evaluate their antioxidant activities by various methods, and their anti-inflammatory activities using in vitro biological methods such as membrane stabilization, protease inhibition, and anti-lipoxygenase activity. In order to be able to understand the mechanism of action, a correlation was also made between the antioxidant activity and the anti-inflammatory effectiveness of the analyzed species.

## 2. Results

### 2.1. Determination of Phytochemical Constituents of C. Sieberiana and P. Thonningii Extracts

**Figure 1:**
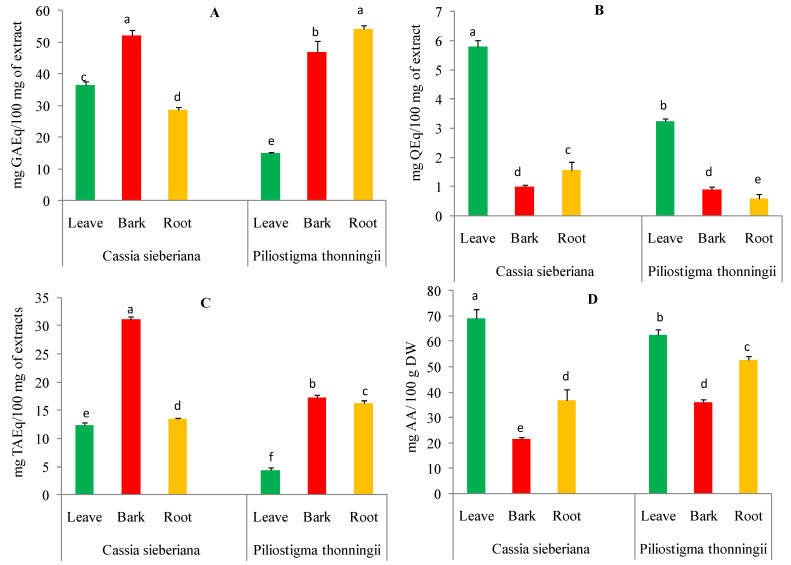
Content of polyphenols (**A**), flavonoids (**B**), condensed tannins (**C**), and vitamin C (**D**) in aqueous extracts of *C. sieberiana* and *P. thonningii* organs. Error bars represent ± standard deviation (SD) of three replicates. Means that do not share the same letter (a, b, c, d, e, f) in the same graph are significantly different by Tukey’s tests (*p* < 0.05).

The total content of polyphenols in the aqueous extracts of *C. sieberiana* and *P. thonningii* from different plant parts showed a large variation. The polyphenol content ranged between 15.07 and 54.06 mg of gallic acid equivalents per 100 milligrams of extract (mg GAEq/100 mg of extract). As shown in Figure 1A, all aqueous extracts from different organs of the two analyzed plants showed significant differences (*p* < 0.05). The highest content was identified in aqueous extracts of *C. sieberiana* bark (52.13 ± 1.62 mg GAEq/100 mg of extract) and *P. thonningii* roots (54.06 ± 1.18 mg GAEq/100 mg of extract) compared to those from other plant parts.

The total flavonoid content of aqueous extracts from *C. sieberiana* and *P. thonningii* plants ranged between 0.58 and 5.80 mg of quercetin equivalents per 100 milligrams of extract (mg QEq/100 mg of extract) (Figure 1B). The highest amounts of flavonoids were recorded for aqueous leaf extracts of *C. sieberiana* (5.80 mg QEq/100 mg of extract) and *P. thonningii* (3.23 ± 0.09 mg QEq/100 mg of extract) compared to other parts of the analyzed plants.

The tannin content of the aqueous extracts of the different parts of the analyzed plants significantly varied from 4.32 to 31.14 mg tannic acid equivalents per 100 milligrams of extract (mg TAEq/100 mg of extract). As shown in Figure 1C, the highest tannin contents were obtained for the aqueous bark extracts of *C. sieberiana* (31.14 ± 0.51 mg TAEq/100 mg of extract) and *P. thonningii* (17.30 ± 0.46 mg TAEq/100 mg of extract).

The vitamin C content of aqueous extracts from *C. sieberiana* and *P. thonningii* different plant organs are shown in Figure 1D. The ascorbic acid content varied between 21.45 and 69.11 ± 3.57 mg ascorbic acid per 100 g of dry weight (mg AA/100 g DW). The leaves of the analyzed species were found to contain the highest amount of ascorbic acid 69.11 ± 3.57 mg AA/100 g DW for *C. sieberiana* and 62.53 ± 2.04 mg AA/100 g DW for *P. thonningii*.

### 2.2. HPLC and UHPLC-MS Analysis of C. Sieberiana and P. Thonningii Extracts

From the spectrophotometric analysis of the various classes of compounds, we deduced that the extract from the bark of *C. siberiana* is the richest in polyphenolic compounds, and the other analyzed samples have a low content of flavonoids. Table 1 shows the results of the HPLC-DAD analysis, which highlight the most important components from different parts of the analyzed plants. Significant differences can be seen both between each analyzed anatomic part of the plant and between species.

The extract with the most diverse and rich polyphenol composition is the one from the bark of *C. sieberiana*; in comparison with the other analyzed samples, it does not contain gallic acid, daidzein, and genistein, but instead, it has an increased content of tannic acid, chlorogenic acid, and rutin. The aqueous extract from the bark of *P. thonningii* proved to be less rich in polyphenol compounds. The chemical composition of the leaves has the smallest differences, the *C. sieberiana* species being richer in hydroxycinnamic acids, which were not identified in the roots of this species nor the leaves and bark of the *P. thonningii* species.

By the UHPLC-MS/MS method, we also proved that the aqueous bark extracts of both species, *C. sieberiana* and *P. thonningii*, presented a rich and varied chemical composition. Thus, the presence of chlorogenic acid in the extracts of the *C. sieberiana* species and vitexin in *P. thonningii* was highlighted.

Chlorogenic acid, which was identified in the aqueous extract from the bark of *C. sieberiana* species, is a biologically active polyphenol that is part of the hydroxycinnamic acids compound class, distributed in many plants, fruits, and vegetables. The compound is widely used in many fields, such as medicine, food, healthcare, and the chemical industry, as it is the main active ingredient of many preparative mixtures in traditional Chinese herbs due to its antioxidant, anti-inflammatory, cardiovascular, hepatoprotective, renoprotective, and antidiabetic properties [34,35]. Chlorogenic acid was identified at the retention time of 12.51 as one of the most intensive peaks and its main MS/MS fragments were m/z 354 (chlorogenic acid) and m/z 112.

In addition, vitexin, a flavone apigenin glycoside found in various foods and medicinal plants, was highlighted in the bark extracts of the species *P. thonningii.* It has a variety of pharmacological effects, including antioxidant, anticancer, anti-inflammatory, and neuroprotective effects [36]. The retention time at which this compound was identified was 16.59, and its main MS/MS fragments were m/z 112, m/z 146, and m/z 174.

### 2.3. In Vitro Antioxidant Activities

In the present study, the scavenging activity of DPPH (2,2-diphenyl-1 picrylhydrazyl) in the aqueous extracts was evaluated, and the IC50s of the samples were also determined (Table 2). The IC50 of different parts of *C. sieberiana* and *P. thonningii* extracts ranged from 13.45 to 72.48 µg/mL, and the best antioxidant activities were observed for the aqueous extracts of the bark (13.45 ± 0.10 µg/mL) of *P. thonningii*, followed by the aqueous extracts of the leaves (21.98 ± 0.19 µg/mL), and then the bark of *C. sieberiana* (22.20 ± 0.35 µg/mL).

The ABTS (2,2’-azino-bis(3-ethylbenzothiazoline-6-sulphonic)) radical scavenger test IC50 values of the extracts varied considerably, from 1.83 ± 0.34 to 26.90 ± 4.41µg/mL. The best radical scavenging activities in the ABTS tests were observed for the aqueous extracts of the roots (1.83 ± 0.34 µg/mL) and bark (1.91 ± 0.26 µg/mL) of *C. sieberiana,* as well as for the bark (5.96 ± 0.28 µg/mL) and roots (6.36 ± 0.20 µg/mL) of *P. thonningii.* All these extracts had similar IC50s (Table 1). The total antioxidant capacity for aqueous extracts of plant parts of *C. sieberiana* and *P. thonningii* demonstrates a lower total antioxidant capacity than vitamin C (44.95 mg AAE/g DW). The highest values of total antioxidant capacity were observed in *P. thonningii* roots (29.68 ± 1.48 mg AAE/g DW) and bark (28.03 ± 0.25 mg AAE/g DW) and the bark of *C. sieberiana* (27.55 ± 1.89 mg AAE/g DW) (Table 2).

### 2.4. In Vitro Anti-Inflammatory Activities

The anti-lipoxygenase activities of aqueous extracts of *C. sieberiana* and *P. thonningii* were evaluated and the results are presented in Table 2. All the extracts of different parts of *C. sieberiana* and *P. thonningii* showed good to moderate enzyme inhibition; IC50 values ranged from 13.04 to 38.07 µg/mL. The bark extracts of *C. sieberiana* (IC50 = 13.04 ± 1.99 µg/mL) and leaf extracts of *P. thonningii* (IC50 = 16.20 ± 1.82 µg/mL) showed similar activities and better anti-lipoxygenase activity compared to others tested extracts. 

In this study, aqueous extracts from the organs of *C. sieberiana* and *P. thonningii* effectively prevented protein denaturation, and significant differences were observed between the aqueous extracts of the two studied plants (*p* < 0.05). The IC50 ranged from 75.74 ± 1.07 µg/mL to 200.16 ± 2.63 µg/mL. The leaves of the two studied species, compared to the other plant parts, showed the best protein denaturation activity (*C. sieberiana* (IC50 = 75.74 ± 1.07 µg/mL) and *P. thonningii* (IC50 = 78.07 ± 0.05 µg/mL)).

All aqueous extracts of *C. sieberiana* and *P. thonningii* protected red blood cells against hemolysis, as shown in Table 3. IC50s ranged from 48.32 ± 6.39 to 67.43 ± 7.36 µg/mL. Thus, the stabilizing potential of the erythrocyte membrane for leaves (IC50 = 48.32 ± 6.39 µg/mL), bark (IC50 = 51.10 ± 0.97 µg/mL), and roots (IC50 = 50, 15 ± 5.23 µg/mL) of *C. sieberiana*, as well as for leaves (IC50 = 53.80 ± 0.75 µg/mL) of *P. thonningii* demonstrated relatively strong and similar anti-inflammatory potentials.

### 2.5. Correlations between Antioxidant and Anti-Inflammatory with Phytochemicals

The statistical correlation between phytochemicals and antioxidant values is shown in Table 4. TAC scavenging activity was strongly correlated with total polyphenol (r = 0.960; *p* < 0.05) and tannin (r = 0.701; *p* < 0.05) contents of aqueous extracts of *C. sieberiana* and of *P. thonningii*. ABTS activity was significantly negatively correlated with the content of total polyphenols (r = −0.679; *p* < 0.05) and tannins (r = −0.702; *p* < 0.05). However, the DPPH test was weakly correlated with phytochemical compounds.

Concerning protein denaturation, the inhibition activity was significantly negatively correlated with vitamin C (r = −0.953; *p* < 0.05) and flavonoids (r = −0.729; *p* < 0.05), and positively correlated with tannins (r = 0.698; *p* < 0.05) and weakly positively correlated withpolyphenols (r = 0.455; *p* < 0.05) (Table 4). During the membrane stabilizing activity, the anti-lipoxygenase activity reached a weak correlation with the groups of phytochemicals measured.

## 3. Discussion

*C. sieberiana* and *P. thonningii* are two plants used in traditional medicine in the Hauts-Bassins region of Burkina Faso to treat hepatitis and inflammatory pathologies [21,37]. The present study evaluated the in vitro antioxidant and anti-inflammatory properties of the different parts (leaves, bark, and root) of these two plants. Several secondary metabolites are produced by plants to ensure their protection, communication, and adaptation to the environment. The diversity of these metabolites, endowed with structural variability, gives them many biological properties [38]. In this study, the total polyphenols, total flavonoids, condensed tannins, and vitamin C of aqueous extracts of *C. sieberiana* and *P. thonningii* were measured. All the aqueous extracts of the different parts of the two plants studied have different quantities of polyphenols, flavonoids, condensed tannins, and vitamin C. These results are in agreement with those in the literature [39,40]. The content of total polyphenols, total flavonoids, condensed tannins, and vitamin C varied with different parts (leaves, stem bark, and root) of the two studied plants (Figure 1). This variability in total polyphenols, total flavonoids, condensed tannins, and vitamin C could be explained by the fact that certain factors, such as genetic, geographical, and climatic factors, have a strong influence on the content of plant phytochemical compounds [41]. Several studies have reported that phenolic compounds and vitamin C have antioxidant and anti-inflammatory properties [37,42]. 

### 3.1. In Vitro Antioxidant Activities

Polyphenols have one or more aromatic benzene rings with mono- or poly-hydroxyl groups in their structure. In addition, these polyphenols have electron or hydrogen donating properties as well as metal reducing and chelation abilities [43]. Previous studies have demonstrated a positive correlation between oxidative stress and the evolution of diseases such as inflammatory pathologies. However, antioxidants are able to reduce this stress, leading to disease prevention [44]. Due to the different complexities in the contents of natural compounds and different reactive oxygen species, several methods are used to assess the antioxidant activities of plant extracts [45]. In this study, three methods (DPPH, ABTS, and TAC) were used to investigate and compare the antioxidant power of *C. sieberiana* and *P. thonningii,* extracts (leaves, stem bark, and root). The statistical correlation between phytochemicals and antioxidant activity was determined and presented in Table 4.

The antioxidant activity of plant extracts is generally attributed to the presence of antioxidants such as phenolic compounds [45]. The results showed that the aqueous extracts of the different parts (leaves, stem bark, and root) of *C. sieberiana* and *P. thonningii* varied in their abilities to scavenge DPPH free radicals (Table 2). According to the classification made by Blois, the extracts with an IC50 < 50 µg/mL are very powerful antioxidants, IC%, values of 50–100 µg/mL belong to powerful antioxidants, 101–150 µg/mL denote medium antioxidants, and an IC50 > 150 µg/mL belongs to weak antioxidants [46]. Based on the IC50 value of DPPH scavenging activity, the aqueous extracts of the leaves (21.98 ± 0.19 µg/mL), bark (22.20 ± 0.35 µg/mL), and roots (31 ± 0.38 µg/mL) of *C. sieberiana* and the bark (13.45 ± 0.10 µg/mL) of *P. thonningii* are very powerful antioxidants. The aqueous extracts of leaves (72.48 ± 0.19 µg/mL) and roots (55.99 ± 0.49 µg/mL) of *P. thonningii* are powerful antioxidants. However, the aqueous extracts of the bark (IC50 = 13.45 ± 0.10 µg/mL) of *P. thonningii*, followed by the aqueous extracts of the leaves (21.98 ± 0.19 µg/mL) and the bark (22. 20 ± 0.35 µg/mL) of *C. sieberiana* demonstrated better DPPH free radical neutralizing activity (Table 1). A weak correlation was found between antioxidant activity and total polyphenols (r = −0.481; *p* < 0.05), condensed tannins (r = −0.585; *p* < 0.05), and vitamin C (r = 0.487; *p* < 0.05) (Table 4). This variability in the DPPH free radical scavenging capacity of the two plant extracts could be linked to the differences in the content of phenolic compounds (polyphenols and condensed tannins) and vitamin C. We could also assume that the antioxidant capacity of the two studied plants does not depend solely on the phenolic compounds present. Certainly, other classes of molecules play an important role in antioxidant activity [39,40]. Previous studies have shown that there is a correlation between the antioxidant activity estimated by the DPPH assay and the levels of phenolic compounds, particularly due to the redox properties of these compounds [37,47,48]. Therefore, phenolic compounds can donate an electron or a hydrogen radical to a DPPH free radical, transforming it into a neutralized stable diamagnetic molecule [49]. 

The ABTS test showed that all extracts have the ability to stabilize the ABTS^•+^ cationic radical by trapping it (Table 2). This reveals the ability of molecules from the two analyzed plants to transfer hydrogen to the radical and to neutralize it [50]. The potential for stabilizing the ABTS^•+^ cationic radical by scavenging varies depending on different parts of the studied plants (Table 2). The aqueous extracts of the different parts of *C. sieberiana* and the aqueous extracts of the bark and roots of *P. thonningii* presented similar and very low IC50s, confirming their strong antioxidant activity (Table 2). ABTS activity was significantly negatively correlated with total polyphenol (r = −0.679; *p* < 0.05) and tannin (r = −0.702; *p* < 0.05) contents, and weakly positively correlated with vitamin C content (Table 4). The high activity of ABTS explains its negative association with the content of phenolic compounds (total polyphenols and condensed tannins) and vitamin C of the aqueous extracts of the two plants. The strong antioxidant activities observed for the aqueous extracts of the bark and roots of *C. sieberiana* and *P. thonningii* would probably be linked to the content of phenolic compounds (total polyphenols and tannins) and vitamin C (Table 2). Several previous studies have reported that phenolic compounds (total polyphenols and tannins) and vitamin C contribute to the antioxidant activities of plants [32,46,51].

The TAC test is an antioxidant test that assesses the ability of an extract to erase a free radical by transferring an electron to it. All the aqueous extracts of the two plants presented a total antioxidant capacity lower than that of ascorbic acid (44.95 ± 0.002 mg AAE/g DW). However, the best TACs were obtained for the aqueous extracts of the roots and bark of *P. thonningii*, followed by the aqueous extracts of the leaves of *C. sieberiana* (Table 2). TAC scavenging activity was strongly correlated with the total polyphenol (r = 0.960; *p* < 0.05) and condensed tannin (r = 0.701; *p* < 0.05) contents of extracts of *C. sieberiana* and *P. thonningii*. The high TAC observed for the root and bark extracts of *P. thonningii* and leaf extracts of *C. sieberiana* could be associated with the content of polyphenolic compounds (total polyphenols and condensed tannins). The total antioxidant capacity of plants is attributed to phenolic compounds, as they possess redox properties allowing them to act as hydrogen donors, reducing agents, singlet oxygen quenchers, or metal chelators [52], results that have been documented in several studies [47,49]. 

### 3.2. Anti-Inflammatory Activities in Vitro

Inflammation can be categorized as acute or chronic and involves a multitude of biochemical events, including the local vasculature, the immune system, and various cell types present in damaged tissues. It is a complex biological response of vascular tissues to aggressive agents such as pathogens, damaged cells, or irritating compounds. Whatever the triggering factor, the mechanisms involved in the inflammatory process are common to all, and the standard signs of inflammation are increased blood flow, increased cellular metabolism, vasodilation, the release of soluble mediators, fluid extravasation, and cell influx [53]. Most of the in vitro analyses of plant species show their anti-inflammatory activity by their ability to inhibit the activity of lipase A2, cyclooxygenase (COX), and lipoxygenase (LOX). However, few plants have been studied for their potential to inhibit other inflammatory mediators, such as interleukins, the production of prostaglandins, thromboxanes, and leukotrienes [54]. 

During inflammation, the phospholipid membrane is hydrolyzed by phospholipase A2 (PLA2), resulting in the release of arachidonic acid (AA). Arachidonic acid is then metabolized by enzymes such as LOX and COX, leading to the release of prostaglandins, thromboxanes, leukotrienes, and hydroxyeicosatetraenoic acids, which are mediators of inflammation [43]. Anti-inflammatories act either by inhibiting the enzymes (PLA2, COX, and LOX) involved in the production of pro-inflammatory mediators, or by preventing the migration and activation of leukocytes at the site of inflammation [49]. In the present study, all aqueous extracts of *C. sieberiana* and *P. thonningii* inhibited LOX with IC50s dependent on the particular part of the plant (Table 3). The best LOX inhibitory activity was obtained with the aqueous extract of the bark (IC50 = 13.04 ± 1.99 µg/mL) of *C. sieberiana*, followed by the aqueous extract of the leaves (IC50 = 16.20 ± 1.82 µg/mL) of *P. thonningii*. Aqueous extracts of *C. sieberiana* and *P. thonningii* possess anti-inflammatory properties because all extracts inhibited LOX. Due to a chemical composition rich in flavonoids and tannins, strong LOX inhibitory activity has been recorded. These results correlated with those of other authors who have highlighted the inhibitory effects of polyphenols (tannins and flavonoids) on LOX [55,56]. Moreover, other previous studies have demonstrated the ability of phenolic compounds, especially flavonoids, to inhibit the biosynthesis of thromboxanes, prostaglandins, leukotrienes, and hydroxyeicosatetraenoic acids by inhibiting phospholipase A2, cyclooxygenase, or lipoxygénase [31,57,58]. 

Protein denaturation is closely linked to the inflammatory process, and the mechanism of action of NSAIDs has been attributed to the inhibition of protein denaturation [59]. Many serine proteinases are present in neutrophil lysosomes. These proteinases play a key role in the development of tissue damage during inflammatory processes. Tissue protection by protease inhibitors has been found [60]. As revealed in Table 3, all extracts of *C. sieberiana* and *P. thonningii* were able to prevent protein denaturation in a dose-dependent manner and significant differences were observed among the studied plant extracts (*p* < 0.05). However, the best protease inhibitory activities were recorded for the aqueous extracts of leaves of *C. sieberiana* (IC50 = 75.74 ± 1.07 µg/mL) and *P. thonningii* (IC50 = 78.07 ± 0.05 µg/mL). In addition, protein denaturation inhibitory activity was significantly negatively correlated with vitamin C (r = −0.953; *p* < 0.05) and flavonoid (r = −0.729; *p* < 0.05) content, and was positively correlated with tannins (r = 0.698; *p*< 0.05) (Table 4). Therefore, the inhibitory activity of *C. sieberiana* and *P. thonningii* extracts could be due to their high content of phenolic compounds (flavonoids and tannins) and vitamin C. Indeed, the different inhibitory denaturation of membrane potential could be explained by the different chemical compositions of the two studied plant extracts. Previous studies have proven that interactions between phenolic compounds (flavonoids and tannins) and proteins improve the thermal stability of proteins [52,55]. Phenolic interactions therefore strongly affect the secondary structure of proteins. Several studies have reported the inhibition of kinases such as protein kinase C, phosphoinositol kinase, phosphatidylinositol kinase, and tyrosine kinase by different types of flavonoids [51]. Flavonoids can regulate protein kinases through the inhibition of transcription factors such as nuclear transcription factor kappa-B (NF-κB). This transcription factor modulates several cytokines, chemokines, and cell adhesion molecules involved in inflammation [43,61]. Other studies have shown that flavonoids modulate the activity of nuclear factor kappa B (IκB) and NF-κB, with a direct impact on cell activation. 

**Figure 2:**
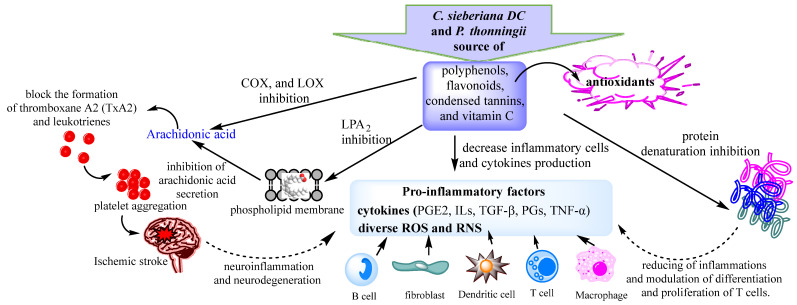
Proposed anti-inflammatory mechanisms of *C. sieberiana* and *P. thonningii* extracts.

The red blood cell membrane is similar to the lysosome membrane; therefore, it remains an excellent model for screening anti-inflammatory compounds. During the inflammatory response, activated neutrophils release lysosomal enzymes at the site of inflammation, promoting inflammation and tissue damage. Extracts that possessed membrane stabilizing activity can act as anti-inflammatories by reducing the release of lysosomal enzymes such as phospholipases, which induce the synthesis of pro-inflammatory mediators. Classical representatives of NSAID drugs have proven their ability to stabilize the lysosome membrane and block the release of lysosomal enzymes. All aqueous extracts of both plants exhibited red blood cell membrane stabilizing activity (Table 3). However, the best anti-inflammatory activities were recorded for the leaf extracts of the two analyzed species (Table 3). Previous studies have shown that polyphenols, in particular flavonoids, can mediate interactions with the phospholipidic cytoplasmic membrane, thereby reducing fluidity and enhancing rigidity [59,62]. These interactions protect the membrane from harmful substances, thus ensuring the physiological function and integrity of the membrane [63]. Several studies have reported that plant-derived flavonoids have anti-inflammatory properties [59,64]. The membrane stabilization potential of the analyzed extracts was weakly negatively correlated with flavonoid content (r = −0.475; *p* < 0.05) and weakly positively correlated with total polyphenol content (r = 0.326; *p* < 0.05). Previous studies have reported a weak correlation between polyphenol content and membrane stabilizing activity (R^2^ = 0.435) [59]. The membrane stabilization potential of the *C. sieberiana* and *P. thonningii* extracts could be due to the high content of phenolic compounds in particular flavonoids, compounds that play an important role in the prevention of the membrane lipid peroxidation against hypotonic and thermal lysis [59,62,65]. These antioxidants, such as flavonoids, polyphenols, and vitamin C, can interact with pro-inflammatory cytokines or can block active sites of COX, LOX, arachidonic acid secretion, and production of prostaglandins and leukotrienes, resulting in a low level of reactive oxygen species and subsequent oxidative stress reduction (Figure 2).

## 4. Materials and Methods

### 4.1. Chemicals and Reagents 

All organic reagents and solvents were purchased from Sigma-Aldrich (Milan, Italy).

### 4.2. Plant Material and Preparation of Aqueous Extracts

#### 4.2.1. Plant Material

**Figure 3:**
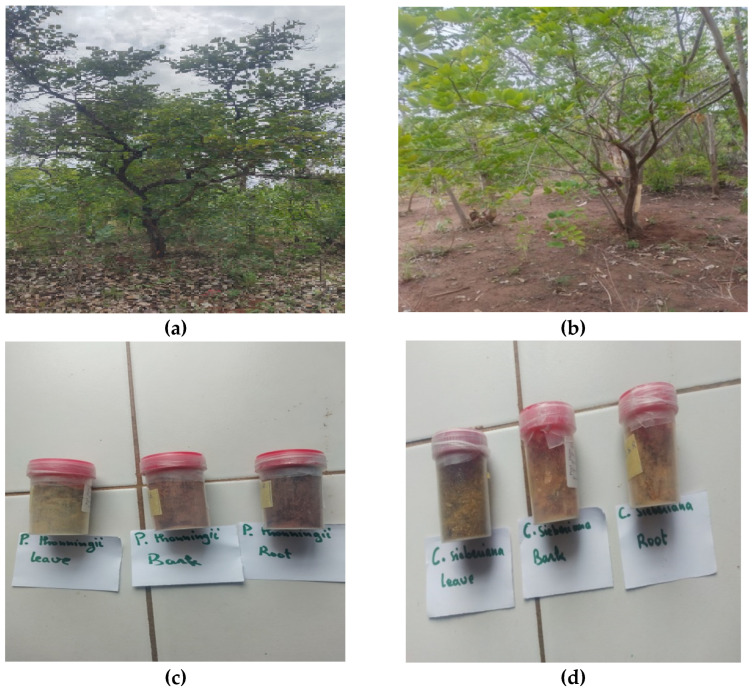
The plants: (**a**) *P. thonningii* and (**b**) *C. sieberiana* from the Hauts-Bassins region, Bobo-Dioulasso, Dienderesso forest. The powder extracts from leaves, roots, and bark of (**c**) *P. thonningii* and (**d**) *C. sieberiana*.

The leaves, roots, and bark of *C. sieberiana* and *P. thonningii* (Figure 3a,b) were harvested in the Hauts-Bassins region, Bobo-Dioulasso, Dienderesso forest. The plants were identified and authenticated at the Department of Botany of NAZI Boni University in Bobo Dioulasso by a botanist, Dr. Ouoba Yempabou Hermann. Specimens were deposited at the Department of Biological Sciences of the Nazi BONI University of Bobo-Dioulasso, Burkina Faso. 

#### 4.2.2. Preparation of Aqueous Extracts 

The harvested plant materials were sorted and washed thoroughly with distilled water to remove dirt and unwanted particles. They were dried under ventilation at room temperature in the laboratory (LARESBA) for two weeks, after which they were crushed to a coarse powder with a mortar. An amount of 30 g of vegetable powder was extracted into 300 mL of distilled water for 30 min to obtain the decoction and filtered using a Watman grade N ° 1 filter. The filtrate was then lyophilized for 10 days, and the obtained material (Figure 3c,d) was used in the following analyses.

### 4.3. Determination of Phytochemical Constituents of Aqueous Extracts

#### 4.3.1. Determination of Total Polyphenols

The content of polyphenols was evaluated by applying the Folin–Ciocalteu method. This method is based on the change of the color from yellow to blue due to the reaction of the Folin–Ciocalteu reagent with phenolic compounds [66,67]. An amount of 125 μL of aqueous extract and 625 μL of Folin–Ciocalteu reagent were mixed, and the mixture was incubated at room temperature for 5 min. After incubation, 500 μL of aqueous sodium carbonate solution (75 mg/mL) was added, and the entire mixture was incubated at room temperature for 2 h. Afterward, the absorbance was recorded at 760 nm, against a control consisting of distilled water, by using a microspectrophotometer. The results were expressed in mg of gallic acid equivalents per 100 milligrams of extract (mg GA Eq/100 mg of extract) using a gallic acid calibration curve. All experiments were performed in triplicate.

#### 4.3.2. Determination of Total Flavonoids

The flavonoid content was determined by applying the method in [68]; 625 μL of aqueous extract (100 ug/mL) and 625 μL of aluminum trichloride (AlCl _3_) were mixed and incubated at room temperature for 10 min. Thereafter, the absorbance was recorded at 415 nm against a control consisting of solvent extraction using a microspectrophotometer. The results were expressed in mg of quercetin equivalents per 100 milligrams of extract (Eq Q mg/100 mg of extract) by a calibration curve with quercetin.

#### 4.3.3. Quantification of Condensed Tannins 

This method measured the ability of vanillin to react with catechin monomers and the terminal units of proanthocyanidins to form a red chromophore complex that absorbs at 500 nm [59,66]. An amount of 500 μL of diluted aqueous extract (1/100) and 1 mL of freshly prepared vanillin sulfur (1 g in 100 mL of 70% sulfuric acid) was mixed and the whole mixture was homogenized and incubated in a water bath at 30 °C for 15 min in the dark. All experiments were performed in triplicate.

#### 4.3.4. Quantification of Ascorbic Acid

The ascorbic acid content of aqueous extracts from the parts of *C. sieberiana* and *P. thonningii* was evaluated by applying the method described in [69]. After dilution, 20 μL of aqueous extract was added to 180 μL of an acidic solution containing 0.1 mg/mL of potassium permanganate (KMnO_4_). The whole mixture was homogenized and incubated in the dark for 5 min. The absorbance of the extracts was read at 530 nm using a microplate reader. In this test, ascorbic acid consumes potassium permanganate (purple solution), causing a decrease in absorbance at 530 nm. All experiments were performed in triplicate.

### 4.4. HPLC and UHPLC-MS Analysis of Aqueous Extracts

Photodiode array high performance liquid chromatography (HPLC-DAD) studies were performed using an L-3000 (RIGOL TECHNOLOGIES, INC Beijing, China). In this chromatographic analysis, was used a Kinetex column EVO C18 (150 4.6 mm, 5 µm particle size). Solvents used were (A) 0.1% trifluoroacetic acid (TFA) in water and (B) 0.1% trifluoroacetic acid (TFA) in acetonitrile, the gradient elution was from 2% A to 100% B at 30 °C for 60 min. An analytical wavelength of 300 nm was used for detection, according to the literature [69,70]. The column was preceded by a security cartridge. UV–Visible spectra were recorded in the range of 210–520 nm for the following compounds: gallic acid, epicatechin, tannic acid, rutin, naringin, genistein, daidzein, hyperoside, chlorogenic acid, and p-coumaric acid. This is a validated method as described by Wandjou et al. [49].

Identification and quantification of polyphenolic compounds by ultra-high-performance liquid chromatography coupled with high-resolution mass spectrometry (UHPLC-MS).

A similar method was used, adapted from the UHPLC-MS method described above, while respecting the parameters for MS. A negative mode HESI (Heated ElectroSpray Ionization) ion source was used for ionization. The ion source parameters were optimized as follows: nitrogen was used as a blanket and the auxiliary gas flow rate was set to 8 and 6 units, respectively. The source heater temperature was set to 300 °C and the capillary temperature was set to 300 °C. Full scan HRMS analysis of the compounds was performed using a Q-Exactiveb Mass Spectrometer. Full scan data in the negative mode were acquired at a resolving power of 70,000 FWHM at m/z 200. A scan range of m/z 100–1000 Da was chosen. External calibration was performed using the calibration solution in both positive and negative modes. 

### 4.5. In Vitro Antioxidant Activities

#### 4.5.1. Free Radical Scavenging Activities of 2,2-Diphenyl-1 Picrylhydrazyl (DPPH)

The antioxidant effects of the tested extracts were evaluated by DPPH radical scavenging in a 96-well plate, with 100 μL of 2,2-diphenyl-1-picryhydrazyl (DPPH) solution added to 100 μL of the extract [38,71]. The absorbance of the samples was measured at 517 nm (Tecan Pro 200 multi-well plate reader) after 20, 35, and 50 min of incubation at room temperature. The control sample used was the solvent mixed with DPPH solution. Trolox was used as a positive control. The percentage of DPPH inhibition was calculated using the following formula:

$$\%\;Inhibition=\frac{\left(Absorbance\;of\;Control-Absorbance\;of\;Sample\right)}{\left(Absorbance\;of\;Control\right)}\times 100$$

#### 4.5.2. ABTS Free Radical Scavenging Activities

The ABTS^•^ test was performed according to the microplate reader assay [72,73]. Briefly, 100 μL of aqueous extract was mixed with 100 μL of ABTS^+^ reagent, and the absorbance was read at 760 nm at different time intervals. The percentage of ABTS free radical scavenging activities was calculated using the following formula:

$$\%\;Inhibition=\frac{\left(Absorbance\;of\;Control-Absorbance\;of\;Sample\right)}{\left(Absorbance\;of\;Control\right)}\times 100$$

#### 4.5.3. Total Antioxidant Capacity (TAC)

This test is based on the reduction of Mo (VI) to Mo (V) and the subsequent formation of a green complex of Mo (V) at acidic pH levels [74]. An amount of 0.1 mL of aqueous extract was mixed with 1 mL of specific reagent solution. They were then incubated at 95 °C for 90 min. After cooling the mixture to room temperature, the absorbance of each solution was measured at 695 nm. The total antioxidant capacity is expressed in ascorbic acid equivalents. All experiments were performed in triplicate.

### 4.6. In Vitro Anti-Inflammatory Activities 

#### 4.6.1. Anti-Lipoxygenase Activity

The test was performed according to the method described by [62,64], with some modifications. The reaction mixture (200 μL) consisted of 160 μL of sodium phosphate buffer (pH 8.0), 10 μL of aqueous extract at various concentrations (10 to 50 μg of extracts were dissolved in Tris buffer (pH 7.4), and 20 μL of lipoxygenase enzyme. The mixture was incubated for 10 min at 25 ° C. The reaction was initiated by the addition of 10 μL of linoleic acid solution as substrate. The mixture was then incubated for 6 min at 25 °C, and the absorbance of the mixture was read at 234 nm in the microplate reader (Tecan Pro 200). All experiments were performed in triplicate. The inhibition percentage was calculated by the following formula:

$$\%\;Inhibition=\frac{\left(Absorbance\;of\;Control-Absorbance\;of\;Sample\right)}{\left(Absorbance\;of\;Control\right)}\times 100$$

#### 4.6.2. Anti-Proteinase Action

A slightly modified test was performed according to the method described in the literature [65]. Aqueous extract (0.5 mL) was added at various concentrations (100 to 500 pg dissolved in 20 mM Tris-HCl buffer (pH 7.4)) with trypsin solution. The whole mixture was incubated at 37 °C for 5 min and afterward, 0.3 mL of 1.5% (G/V) casein was added. The whole reaction mixture was incubated again for 20 min, and to complete the reaction 0.2 mL of perchloric acid 70% was added. The reaction mixture was then centrifuged at 5000 rpm for 5 min and the absorbance of the supernatant was read at 210 nm in a microplate reader (Tecan Pro 200). The inhibition percentage was calculated by the following formula:

$$\%\;Inhibition=\frac{\left(Absorbance\;of\;Control-Absorbance\;of\;Sample\right)}{\left(Absorbance\;of\;Control\right)}\times 100$$

### 4.7. Membrane Stabilization Test 

For the preparation of red blood cells for analysis, were followed the steps of the method described in [60,75]. The reaction mixture consisted of 800 µL of test extract at different concentrations (100 to 500 µg of extract were dissolved in 1 mL of normal saline) and 800 µL of 10% red blood cell suspension, for control, was used saline solution. Diclofenac sodium was taken as a standard medicine. All tubes containing the reaction mixture were incubated at 56 °C for 30 min. The reaction mixture was cooled to room temperature, centrifuged at 2500 rpm for 5 min, and the absorbance of the supernatant was read at 560 nm in a microplate reader (Tecan Pro 200). The inhibition percentage was calculated by the following formula:

$$\%\;Inhibition=\frac{\left(Absorbance\;of\;Control-Absorbance\;of\;Sample\right)}{\left(Absorbance\;of\;Control\right)}\times 100$$

### 4.8. Statistical Analyzes

All measurements were performed in triplicate and the results were expressed as mean ± standard error using GraphPad Prism 9.1.0. and Excel 2010 software. A one-way analysis of variance (ANOVA One way) was performed with Minitab version 19.1.0 and a *p* value of less than 0.05 was found to be significantly different. Linear regression analysis was used to calculate the IC50 value.

## 5. Conclusions

The results of the present study, consisting of an in vitro analysis of the antioxidant and anti-inflammatory capacities of different aqueous extracts (leaves, bark, and roots) of *C. sieberiana* and *P. thonningii*, demonstrated the biological properties of these species that have been—and can be—used to treat various inflammatory diseases. This is due to a chemical composition that has been highlighted with the help of several methods of dosing and identification of compounds. Most interestingly, compounds with antioxidant and anti-inflammatory action were identified in the bark of the plants. The results obtained for anti-inflammatory activities suggested that vitamin C, chlorogenic acid, and vitexin were the main providers of this anti-inflammatory effect. The antioxidant activities were higher or lower depending on the part of the plant analyzed, due to the diverse chemical composition. The results and findings obtained could validate the traditional use of these plants and their biological potential, and work is being done to incorporate them into various forms that are easier to use. 

## Figures and Tables

**Table 1:** Chemical composition of *C. sieberiana* and *P. thonningii* extracts (expressed as µg/mL and ± SD).

Compounds	RT *	*C. sieberiana*			*P. thonningii*		
		Root	Bark	Leaves	Root	Bark	Leaves
**Hydroxybenzoic acids**							
Gallic acid	5.9	n.d.	n.d.	18.029 ± 0.41	26.744 ±1.04	216.069 ± 1.59	n.d.
Epicatechin	23.9	27.393 ± 0.78	26.867 ± 0.59	n.d.	n.d.	n.d.	n.d.
Tannic acid	24.3	n.d.	1077.675 ± 1.60	12.27 ± 1.33	10.478 ± 0.81	11.638 ± 0.55	n.d.
**Flavons**							
Rutin	31.4	n.d.	623.84 ± 0.70	490.517 ± 1.04	31.138 ± 0.66	n.d.	n.d.
Naringin	11.3	27.67 ± 0.96	107.352 ± 0.26	816.968 ± 1.11	545.117 ± 1.95	n.d.	457.14 ± 1.04
Genistein	22.7	421.376 ± 1.23	n.d	n.d.	n.d.	n.d.	n.d.
Daidzein	33.6	148.362 ± 2.45	n.d	n.d.	n.d.	118.029 ± 0.38	n.d.
Hyperoside	6.9	80.296 ± 0.77	484.07 ± 0.84	n.d.	n.d.	n.d.	n.d.
**Hydrocinnamic acids**							
Chlorogenic acid	22.3	n.d.	705.412 ± 2.67	n.d.	n.d.	n.d.	23.435 ± 0.59
p-Coumaric acid	28.9	1.757 ± 0.29	56.36 ± 1.01	n.d.	n.d.	n.d.	0.217 ± 0.92
Total		706.854 ± 6.48	3081.576 ± 7.67	1337.784 ± 3.89	613.477 ± 4.45	345.736 ± 2.52	480.792 ± 2.55

RT * means retention time, n.d. means not detected.

**Table 2:** Antioxidant activity of aqueous extracts of *C. sieberiana* and *P. thonningii* determined by DPPH, ABTS, and TAC assays.

Plants	Parts of the Plant	DPPH (IC50 µg/mL)	ABTS (IC50 µg/mL)	TAC (mg AAE/g DW)
*Cassia sieberiana*	Leaves	21.98 ± 0.19 ^d^	5.96 ± 1.20 ^b^	25.63 ± 0.58 ^c^
	Bark	22.20 ± 0.35 ^d^	1.91 ± 0.26 ^b^	27.55 ± 1.89 ^bc^
	Root	31.00 ± 0.38 ^c^	1.83 ± 0.34 ^b^	18.90 ± 0.76 ^d^
*Piliostigma thonningii*	Leaves	72.48 ± 0.19 ^a^	26.90 ± 4.41 ^a^	14.12 ± 0.30 ^e^
	Bark	13.45 ± 0.10 ^e^	6.36 ± 0.20 ^b^	28.03 ± 0.25 ^bc^
	Root	55.99 ± 0.49 ^b^	5.96 ± 0.28 ^b^	29.68 ± 1.48 ^b^
Trolox		10.57 ± 0.0019	32.56 ± 0.0016	-
Vitamin C		-	-	44.95 ± 0.002 ^a^

The IC50 was obtained using the regression equation. Mean values (*n* = 3) ± SD that do not share the same letter (a, b, c, d, e) in the same column are significantly different according to Tukey’s tests (*p* < 0.05)**.**

**Table 3:** In vitro anti-inflammatory activity of aqueous extracts of *C. sieberiana* and *P. thonningii*.

Plants	Parts of the Plant	Anti-Lipoxygenase Activity (IC50 µg/mL)	Protease Inhibitory Activity (IC50 µg/mL)	Membrane Stabilization Activity (IC50 µg/mL)
*Cassia sieberiana*	Leaves	31.53 ± 7.93 ^a^	75.74 ± 1.07 ^e^	48.32 ± 6.39 ^c^
	Bark	13.04 ± 1.99 ^d^	200.16 ± 2.63 ^a^	51.10 ± 0.97 ^bc^
	Root	38.07 ± 2.62 ^a^	188.02 ± 0.85 ^c^	50.15 ± 5.23 ^c^
*Piliostigma thonningii*	Leaves	16.20 ± 1.82 ^cd^	78.07 ± 0.05 ^e^	53.80 ± 0.75 ^bc^
	Bark	24.79 ± 2.61 ^b^	195.87 ± 0.83 ^b^	67.43 ± 7.36 ^a^
	Root	22.01 ± 1.36 ^bc^	117.77 ± 3.63 ^d^	58.87 ± 1.80 ^b^
Aspirin (acetylsalicylic acid)		-	160.20 ± 0.02	-
Indomethacin Diclofenac sodium		45.12 ± 0.014	-	-
		-	-	68.78 ± 0.07

The IC50 was obtained using the regression equation. Mean values (*n* = 3) ± SD that do not share the same letter (a, b, c, d, e) in the same column are significantly different according to Tukey’s tests (*p* < 0.05)**.**

**Table 4:** Pearson (r) correlation of phytochemicals with antioxidant and anti-inflammatory activities.

		DPPH	ABTS	TAC	Anti-Lipoxygenase Activity	Protease Inhibitory Activity	Membrane Stabilization Activity
Polyphenols	r	−0.481	−0.679	0.960	−0.199	0.455	0.326
	*P*	0.043	0.015	0.000	0.429	0.058	0.187
Flavonoids	r	0.016	0.312	−0.317	0.269	−0.729	−0.475
	*P*	0.949	0,324	0.316	0.284	0.001	0.046
Tannins	r	−0.585	−0.702	0.701	−0.310	0.698	0.032
	*P*	0.011	0.011	0.011	0.210	0.001	0.900
Vitamin C	r	0.487	0.583	−0.324	0.173	−0.953	−0.187
	*P*	0.040	0.047	0.305	0.493	0.000	0.481

## Data Availability

Not applicable.
